# “I cry every day and night, I have my son tied in chains”: physical restraint of people with schizophrenia in community settings in Ethiopia

**DOI:** 10.1186/s12992-017-0273-1

**Published:** 2017-07-11

**Authors:** Laura Asher, Abebaw Fekadu, Solomon Teferra, Mary De Silva, Soumitra Pathare, Charlotte Hanlon

**Affiliations:** 1 Centre for Global Mental Health, Department of Population Health, London School of Hygiene and Tropical Medicine, London, UK; 2 Department of Psychiatry, School of Medicine, College of Health Sciences, Addis Ababa University, Addis Ababa, Ethiopia; 3 Centre for Affective Disorders, Department of Psychological Medicine, Institute of Psychiatry, Psychology and Neuroscience, King’s College London, London, UK; 4 Department of Epidemiology, Harvard T. H. Chan School of Public Health, Harvard University, Boston, USA; 5 Centre for Mental Health Law and Policy, Indian Law Society, Pune, India; 6 Centre for Global Mental Health, Health Services and Population Research Department, Institute of Psychiatry, Psychology and Neuroscience, King’s College London, London, UK

**Keywords:** Schizophrenia, Ethiopia, Human rights, Physical restraint, Mental disorders, Community mental health services

## Abstract

**Background:**

A primary rationale for scaling up mental health services in low and middle-income countries is to address human rights violations, including physical restraint in community settings. The voices of those with intimate experiences of restraint, in particular people with mental illness and their families, are rarely heard. The aim of this study was to understand the experiences of, and reasons for, restraint of people with schizophrenia in community settings in rural Ethiopia in order to develop constructive and scalable interventions.

**Methods:**

A qualitative study was conducted, involving 15 in-depth interviews and 5 focus group discussions (*n* = 35) with a purposive sample of people with schizophrenia, their caregivers, community leaders and primary and community health workers in rural Ethiopia. Thematic analysis was used.

**Results:**

Most of the participants with schizophrenia and their caregivers had personal experience of the practice of restraint. The main explanations given for restraint were to protect the individual or the community, and to facilitate transportation to health facilities. These reasons were underpinned by a lack of care options, and the consequent heavy family burden and a sense of powerlessness amongst caregivers. Whilst there was pervasive stigma towards people with schizophrenia, lack of awareness about mental illness was not a primary reason for restraint. All types of participants cited increasing access to treatment as the most effective way to reduce the incidence of restraint.

**Conclusion:**

Restraint in community settings in rural Ethiopia entails the violation of various human rights, but the underlying human rights issue is one of lack of access to treatment. The scale up of accessible and affordable mental health care may go some way to address the issue of restraint.

**Trial registration:**

Clinicaltrials.gov NCT02160249 Registered 3rd June 2014.

## Background

People with mental illness commonly experience human rights violations in both community settings and mental health facilities [1]. Since it was adopted by the United Nations in 2006, the Convention on the Rights of Persons with Disabilities (CRPD) has been ratified by 166 countries. The Convention provides a legal framework for protecting and promoting the human rights of some of the most vulnerable groups in society [2]. The need to tackle human rights violations has been described as the most important reason for scaling up mental health services in low and middle-income countries (LMIC) [3]. Providing care for people with mental illness in settings with few human and financial resources is a challenging task in itself. The question of how to adequately and appropriately address human rights violations in the process of scaling up mental health services is of critical importance [1, 4–6].

Amongst mental disorders, schizophrenia has been identified as a global priority [5]. Whilst lifetime prevalence is relatively low (4/1000 population globally [7] and 4.7/1000 in Ethiopia [8]), the often severe and chronic nature of schizophrenia means it can have a catastrophic impact on individuals and their families, expressed in high levels of mortality (over three times that of the general population in Ethiopia [9]), disability [10] and economic burden [11]. Insufficient service provision is one of the reasons why 90% of people with schizophrenia in rural Ethiopia do not access formal care [12], a treatment gap reflected in other LMIC [13]. Instead, the majority of the care burden falls on family members [11, 14], who undertake this role without any social security provision or formal community-based care.

There are several accounts from sub-Saharan Africa and Asia of the restraint and confinement of people with mental illness by family members [6, 15–21]. The Indonesian government estimates there are 18,800 people with mental illness across Indonesia who are physically restrained in the community [21]. The extent, nature and experience of restraint in community settings in Ethiopia has not been evaluated. However, there is anecdotal evidence that it is a common experience amongst people with schizophrenia in this setting [22]. The academic and human rights advocacy literature typically focus on physical abuse and restraint within institutions, including psychiatric hospitals and traditional healing centres [1, 4, 20, 23–26], sometimes neglecting restraint in the family home. This represents an important omission given that in many countries, most people with mental illness will never access any institutional care; instead they live in the community and are cared for primarily by family members.

Whilst the link between restraint in the community and lack of access to anti-psychotic medication has been clearly made [3, 6, 15], there is also a tendency to assume that restraint is related to misconceptions about the cause of mental illness (typically that schizophrenia is related to spirit possession) and associated stigma [17, 18, 23, 24]. Understanding and contextualising why restraint occurs in a particular setting is important [6]. The factors leading to human rights abuses are often complex and unlikely to have simple solutions. An incomplete understanding of the reasons for restraint in community settings may lead to the development of inappropriate and ineffective interventions and legislation to address the problem. In particular greater attention should be paid to the voices of those who are restrained, which have historically been unheard (with some exceptions e.g. Read et al. [6]). Alongside this, accounts from those who are effecting and condoning restraint within the community are needed for a complete understanding of the problem. The aim of this study was to understand the experiences of, and reasons for, restraint of people with schizophrenia in community settings in rural Ethiopia in order to develop constructive and scalable interventions to reduce the use of restraint.

## Methods

### Setting

The study was conducted in Sodo district and the adjacent districts around Butajira town, in the Gurage administrative zone of the Southern Nations, Nationalities and Peoples’ Region of Ethiopia. Sodo is 100 km from Addis Ababa and Butajira is 130 km away. The majority of the population live in rural areas. The topography is variable, encompassing both cool mountainous areas and lowlands with higher temperatures. Most of the population work as subsistence farmers and live in mud and straw houses. Around 51% of the population is estimated to be literate [27]. In the Butajira area, the majority are Muslim, whilst in Sodo district the majority are Orthodox Christian. Biomedical care, traditional healers and holy water are utilised for mental health problems in this area. Holy water, which is believed to have curative properties, is accessed by both Christians and Muslims at specific sites associated with the Ethiopian Orthodox Church. There are various other types of traditional healers, ranging from herbalists to *tanqway* (sorcerers)*,* who use tinctures, animal sacrifices and rituals, and *debtera*, who are priests believed to have magical powers [28].

There is a psychiatric nurse-led outpatient clinic in Butajira hospital, through which the participants with schizophrenia and caregivers were identified in this study. At the time the study was conducted there were no mental health services in Sodo district. People with mental health problems in this area either had to attend the Butajira clinic or otherwise travel 100 km to Ammanuel psychiatric hospital in Addis Ababa. Sodo district is the setting for the Ethiopian arm of the PRogramme for Improving Mental healthcarE (PRIME) project. PRIME is a five-country research consortium that aims to generate evidence on the integration and scale up of mental health into primary and maternal care settings [29]. As part of PRIME a scalable mental health care plan was developed and implemented in Sodo district across community, facility and district healthcare levels [30].

### Data collection

From July to September 2013, five focus group discussions (FGDs) with a total of 35 participants and 15 in-depth interviews (IDIs) were conducted with people with schizophrenia, their caregivers, community and religious leaders, health extension workers (community health workers), community-based rehabilitation (CBR) workers and primary care staff (see Table 1). The primary aim of the IDIs and FGDs was to determine the acceptability and feasibility of CBR for people with schizophrenia in this setting. This formed part of the Rehabilitation Intervention for people with Schizophrenia in Ethiopia (RISE) project. The results of this formative work have been published previously [31]. A secondary aim of the IDIs and FGDs was to explore the issue of restraint in community settings, with a view to understanding the best way to intervene. Hence, the topic guides covered (i) current problems and needs (ii) experiences or awareness of restraint and (iii) potential ways to address restraint.

**Table 1:** IDI and FGD participants

	People with schizophrenia	Caregivers	Community leaders	CBR workers, primary care staff and health extension workers
Number of IDI participants	4	2	7	2
Number of FGD participants	0	15	0	20
Age categories				
<25	0	0	0	8
25–34	2	8	0	12
35–44	0	2	1	2
45–59	2	3	3	0
60 and above	0	4	3	0
Gender				
Male	3	9	7	5
Female	1	8	0	17
Education				
Cannot read and write	2	7	0	0
Read and write only	1	2	3	0
Primary	1	7	1	0
Secondary	0	0	1	0
Post-secondary	0	1	2	22
Community leader type				
Kebele chairperson	-	-	1	-
Religious leader (Christian)	-	-	1	-
Religious leader (Muslim)	-	-	1	-
Microfinance head	-	-	1	-
Herbalist	-	-	1	-
Social court chairperson	-	-	1	-
Edir leader	-	-	1	-
Occupation				
Farmer	2	14	1	-
Merchant	1	2	2	-
Unemployed/ pensioner	1	0	1	-
Employed	0	1	3	-
CBR worker	-	-	-	6
CBR supervisor	-	-	-	2
Health extension worker	-	-	-	8
Primary care health officer/nurse	-	-	-	6

Primary care staff and health extension workers were identified through Sodo district health bureau. CBR workers and supervisors were employees at the Rehabilitation And Prevention Initiative against Disabilities (RAPID) project in Adama, which supports children with disabilities. People with schizophrenia and their caregivers were identified through the Butajira psychiatric outpatient clinic. Community leaders were either members of the PRIME community advisory board [30] or were identified through PRIME field workers. Participants were selected purposively to ensure a spread of gender, work experience, type of community leader and functional status of people with schizophrenia. The participants were invited by telephone or face-to-face and all those approached agreed to take part. All participants received modest remuneration (equivalent to US$3) for their time and transportation costs.

The IDIs and FGDs were conducted in Amharic by an Ethiopian psychiatrist (ST) and an Ethiopia PhD student with a psychology MSc (KH). Both had experience in conducting IDIs and FGDs with people with schizophrenia and their caregivers [32–34]. No relationship between the researchers and participants existed in advance. The interviews were conducted at local health centres and private offices. IDIs lasted between 40 and 60 min and FGDs lasted between 60 and 120 min and all were audio-recorded. LA observed the interviews and discussed the content with the interviewers immediately after each one, making hand written notes. The audio-recordings were transcribed in Amharic, and then translated into English. If the translation was ambiguous or included cultural references that required interpretation, LA discussed and clarified the meaning with ST and KH.

### Data analysis

A thematic analysis of the IDIs and FGDs was conducted, using NVivo for Mac software to manage the data. Thematic analysis is a method which sits between a realist approach (in which experiences are described) and a constructionist approach (where experiences are seen to reflect wider discourses operating in society) [35]. An inductive (data driven) approach to identifying themes was employed; we did not consider the data with an a priori coding frame [35]. LA first familiarised herself with all transcripts, noting initial impressions. Two transcripts were independently coded by LA and ST, and a meeting was held to discuss differences and make minor adjustments to the coding scheme. Once a consensus was reached, all manuscripts were indexed by LA using the final coding scheme developed, but also adding additional codes as required by the data. LA collated the codes into potential themes and sub-themes, through seeking repeated patterns of meaning across the dataset [36]. LA created a map of how the themes were related, which was discussed and finalised with ST. Themes and sub-themes were reviewed by checking whether the collated quotes for each theme were coherent, and collapsing or expanding sub-themes as required. LA then reread the full transcripts to check the final thematic framework adequately reflected the totality of the data collected. We summarised and interpreted the themes using a contextualist approach, in that we retained focus on the data and the reported experiences of individuals, but we tried to understand how the broader social context, for example living conditions and access to healthcare, shaped those experiences [35, 37]. Associations between themes and patterns relating to participant characteristics were noted, for example we compared the reports of people with schizophrenia against those of caregivers. Quotes were selected by LA to exemplify each theme and sub-theme.

We were able to examine the validity of emerging themes, and supplement quotations to support themes, using a second qualitative dataset obtained for the PRIME project. The PRIME dataset included 13 IDIs and five FGDs conducted with similar stakeholders to the RISE study [38]. The primary aim of the PRIME qualitative study was to inform development of the district mental health care plan, the results of which have been published elsewhere [30, 39]. In relation to this aim the topic guide enquired about the experience of physical restraint and possible approaches to address restraint.

### Ethics approval and consent to participate

Ethical approval was obtained from the Addis Ababa University College of Health Sciences Institutional Review Board (reference 039/13/PSY) and from the London School of Hygiene and Tropical Medicine Research Ethics Committee (reference 6408). Written informed consent, or a witnessed thumbprint for those who were illiterate, was obtained from all study participants. Prior to conducting the interviews with people with mental illness, capacity to consent to participate in the study was evaluated by a psychiatrist. Consent for publication was obtained from all participants.

## Results

**Fig. 1:**
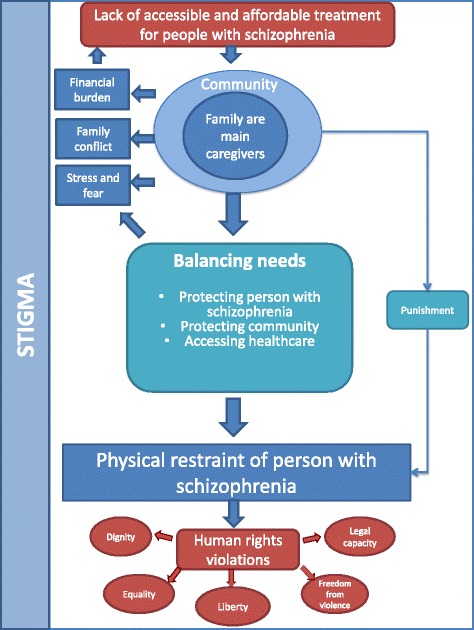
Conceptual model of drivers and consequences of restraint

There were two overarching themes: (1) Experiences and impact of restraint and (2) Reasons for restraint. The specific reasons for restraint, which included the need to protect the person with schizophrenia, to protect people and property and to access health care, were underpinned by heavy caregiver burden, a sense of powerlessness and pervasive stigma towards people with schizophrenia. Figure 1 illustrates the relationship between these themes.

### Experiences and impact of restraint

#### Manner of restraint

Almost all people with schizophrenia and caregivers reported personal experiences relating to restraint. Awareness of the practice of restraint was near universal amongst the community leaders, CBR workers, health extension workers and health centre staff. Only one participant, a community leader, denied the existence of the practice. Respondents were consistent in reporting that restraint takes place in the community, with the individual typically tied up inside the house or in one case tied to a tree. Only one person with schizophrenia reported being restrained at a holy water site. There were several reports of restraint being utilised on the journey to a health facility. There were no reports of restraint in institutional settings; although most of the people with schizophrenia in this rural district would not have had access to psychiatric inpatient care or any other type of institution. The manner of restraint included tying the person’s hands behind their back, the use of handcuffs, chains and in one case an iron bar.

“*I remember this girl with severe mental health problems. We used to see her when we went out together, handcuffed to a tree in her family’s compound. Her mother is an old woman. She did that because the girl used to cause so much trouble.”*

(FGD 05 participant 1, female CBR worker)

#### Patterns of restraint

It was difficult to precisely quantify the typical duration of restraint, though some patterns emerged. Three people with schizophrenia and one caregiver described long periods of restraint, specified in two cases as eight months and two years in duration. One man with schizophrenia reported the issue as follows:

“Interviewer*: How long have you been tied up consecutively?*

Participant*: It is for a long time, I didn’t count it but if I did it is for a long time*

I*: Do they tie you all the time?*

P*: Yes.*”

(IV06, man with schizophrenia)

Participants ‘external’ to the experiences, including the community leaders, community workers and health workers, tended to emphasise the long duration of restraint; however these accounts were often given in general terms without reference to specific individuals. Some caregivers reported a different pattern of restraint, characterised by the individual being restrained then unrestrained for short periods.

“*We don’t put him in chains always. But what can you do when he threatens someone’s life? When we feel like he is doing better, we unchain him. When he tries to attack someone, we chain and shackle him back*.” (PRIME IV15, male caregiver)

Physical violence towards people with mental illness, not occurring in the context of restraint, was less commonly reported by any type of participant. However one male caregiver reported being physically violent towards his son with schizophrenia, and two women with schizophrenia reported being tied up and beaten, including at a holy water site:

“*I: Have you ever been tied?*

*P: You mean there [at the holy water site]?*

*I: Yes*

*P: Like this*

*I: They tied you backwards?*

*P: Yes, when they hit me like this, they carried me and took me in…This is not working now*

*I: Ok were you hit on the forehead?*

*P: Yes*

*I: What did they use to hit you on the forehead?*

*P: He used his hand; his hand was so hard like metal, in the name of the father and the son”*

(IV01, woman with schizophrenia)

#### Perpetrators of restraint

Family members were almost always reported to be the main group instigating the restraint. One woman with schizophrenia explained,

*“I was chained and restrained at home when I got mentally ill for the first time. I was beaten by everyone at home when I tried to leave the house. I don’t remember if I was beaten by people outside of my family. I had people visiting me. They did not beat me. Only my family members who were there to attend me at home beat me when I went out of control” *(PRIME IV16, woman with schizophrenia).

There was one experience of restraint at a holy water site reported by a person with schizophrenia, as described above. However, a holy water priest and a Christian church leader both reported that they do not condone restraint. The holy water priest described how he had released a woman with mental illness from restraint who had been brought to his holy water site. Despite typically being carried out by the family, restraint appeared to be generally condoned and also facilitated by the wider community. There were some reports of families calling on other community members for assistance. One man with schizophrenia reported:

“I: *My father says “tie him up”, he calls people and makes sure they gang up on me and tie me up*

P: *Does he gather people from the locality to tie you?*

I: *They say that I am crazy, he says that I am crazy and they should tie me up*.”

(IV06, man with schizophrenia)

#### Impact of restraint

Some participants, including two people with schizophrenia, spoke of the physical injuries, sometimes permanent, that could result from restraint. Community leaders and community health workers highlighted the negative psychological impact of being restrained, and acknowledged that it could make the illness worse or increase the risk of violence towards others. One religious leader described the potential impact as follows:

“*Throwing them [people with mental illness] in chains and keeping them at home will aggravate their illness. That will make the person hopeless. That will darken their hope.*”

(PRIME IV05, religious leader).

### Reasons for restraint

#### Underpinning themes

There was a strong emphasis amongst caregivers that they are left to shoulder the burden of care alone, often without support from other family members or the wider community. One female caregiver commented:

*“There is no help from a relative, a father, a mother, a sister, a significant person …There is no support for patients, elderly, people who are weak in our country” *(FGD 02 participant 6, female caregiver)

Caregivers supported individuals with schizophrenia in various practical ways, most prominently reminding them to take medication and taking them to healthcare appointments. Some caregivers also reported providing moral support and having a calming influence on their relative. Caregivers, along with health care workers, also described the financial hardship that supporting a relative with mental illness could bring. Several caregivers reported that the burden associated with caring had made them ill themselves or ‘like a patient’.

*"I am thinking of leaving the house, that’s it, I even need divorce…I, that’s it, I am having a hard time when it comes to him, he doesn’t listen to me when I tell him to stop, when I say “this thing is not good”, then my head gets hot, that’s it, I am also sick, that’s it, he doesn’t accept many ideas…I feel bad" *(FGD 02 participant 2, female caregiver)

Caregivers’ decisions about restraint were frequently driven by fear of the consequences of the individual being unrestrained. The overarching impression was that family members often felt hopeless, helpless and that they had no choice but to restrain their relative. Caregivers tried to balance the perceived needs of the individual, the family and the community in making their decision. On the one hand there was a desire to protect the individual from harm, and to help them to access treatment, though respondents did not focus on the treatment preferences of the individual. On the other hand was the need to protect the community. In the middle was the caregiver themselves, often distressed by the task of restraining and the stress of restraining and unrestraining. One female caregiver stated,

“*I cry every day and night. I have [my son] tied in chains; I cry holding his hands and legs.*” (PRIME FGD05, female caregiver).

Another female caregiver explained:

*“He was afraid of being tied, but he was tied. He was stressed out for about 5 days, we tied him, we tied him for 5 days. I was also stressed out along with him.*” (IV02, female caregiver)

A minority expressed the need for proportionality; for these participants restraint was seen as a selective process that is not relevant for all people with mental illness, only those who are aggressive or who have a long duration of untreated illness. Furthermore, several community leaders disapproved of restraint unless they perceived there to be a benefit to the person with schizophrenia. Whilst stigma did not appear to be a sole reason for restraint, stigmatizing attitudes towards people with mental illness seemed to be the backdrop against which these practices were carried out. Amongst some participants, particularly amongst community leaders but also caregivers, there appeared to be an assumption that people with schizophrenia are dangerous and unpredictable.

*“Now someone who has a child with mental illness has killed his friend, his mother, his father, his brother, his sister, his neighbor, he starts fire in a neighbor’s house, he starts fire in a house made of grass. Since this is a big danger, since this affects the society, so the society has to take it seriously” *(FGD 01 participant 4, male caregiver)

In other cases stigma was expressed in the person with schizophrenia being seen as insignificant or lacking usual needs; either being just ‘a patient’, not feeling pain, or being compared to a dog needing to be restrained. One woman with schizophrenia could not pinpoint any rationale for her restraint, stating simply, *“they inflict violence on me because I am a patient, I have nothing else you know.*” (IV01, woman with schizophrenia). A female caregiver suggested that her daughter with schizophrenia did not feel the pain of being beaten and bruised: *“People would beat her up but she doesn’t feel anything. She looks like this [black] cloth you’re wearing; she doesn’t bleed.”* (PRIME FGD05, female caregiver).

### Specific reasons for restraint

#### A means to access healthcare or medication

The use of restraint to transport unwell individuals to holy water, the health centre or to the psychiatric hospital in the capital city so that they could receive treatment was described by several participants. In most cases the implication was that without the restraint it would not have been physically possible to make the journey, and was therefore necessary to access the treatment required to improve the situation. In one case the emphasis was on the individual being unwilling to attend the health facility independently. Although at times this particular man with schizophrenia stressed the unnecessary nature and harmful effects of restraint, he also suggested in some circumstances the outcome of restraint was positive; being restrained enabled him to access the medication which had ultimately improved his illness.

“P: *Another boy who was also ill came and took medication here [at the clinic], then he left saying that ‘I don’t want to take the medication because it is tiring me’ and he does not come here and take the medication, he left. I am the only one who did not leave…The reason why I did not leave is that my parents bring me forcefully, they say that I have to come down and take medication, they tie me up and bring me here [to Butajira]*

I: *So, the fact that there is help from your parents has benefited you.*

P: *It has benefited me a lot, the medication is good for me”*

(IV06, man with schizophrenia).

Two caregivers described using restraint to forcibly administer oral or injectable medication, whilst stressing they considered this action as a last resort. One male caregiver described the situation as:

“*Sometimes, when it is beyond what we can handle, when they [the individual with schizophrenia] refuse to take medication, we tie them and then feed them opening their mouths forcefully after dissolving it in water. Because they are stubborn for a while till they get back to their senses… since there is a situation beyond patience and resistance.” *(FGD01 participant 4, male caregiver)*.*

Across all types of participants, obtaining treatment for mental illness was recommended as the best way to avoid restraint. One caregiver felt that bringing medication to the home would prevent the use of restraint on the journey to the health facility.

#### Protection of the person with schizophrenia

All groups, with the exception of people with schizophrenia, cited fear for the individuals’ safety as a reason for restraint. The tendency of people with schizophrenia to wander off from the house for several days was described in relation to the stress and uncertainty this brought to family members at home. Perceived risks to people with schizophrenia included running away permanently, being injured or hit by a car, being attacked by hyenas, being washed away in a flood, and getting into physical confrontations with others. One female caregiver described her fears as follows:

*“I get very much scared when he goes out, disappears and gets back after four, five days. I ask them what happened to my son, I look everywhere, and now I tied him thinking that even if he dies he should die right here with me. Now he is asking us to untie him, we told him we will but we did not mean it.” *(IV02, female caregiver)

A herbalist and a religious leader both emphasised that restraint for protection should not be a blanket measure for all people with mental illness, but reserved for those who are aggressive or who have the worst symptoms. They also described the difficulties families face in balancing up the needs of people with schizophrenia:

*“Well, even if there is advice [not to restrain people with schizophrenia], if they don’t get tied up, they might cause damage. There will be greater damage, if those are not tied up, the craziest ones, if they are not tied up, they will cause damage, or they might get hit by a car. And they might die being hit by a car, so it is hard both ways.”*

(IV13, Orthodox Christian leader)

Only one participant, a community leader, specifically referred to restraint as a means to reduce the risk of suicide.

#### Protection of other people and property

The threat of physical violence from people with schizophrenia towards others was frequently cited. For some caregivers, this was in the context of the arguments and household conflict, particularly with their spouse. One female caregiver described her constant worry as follows.

*“He will do many things, he will kill people, now he will kill me, I don’t go to sleep before hiding this knife in my house, because, I have no trust, now I don’t have anything to hide from you, I have no trust,”*

(FGD 02 participant 1, female caregiver)

For several participants, this perceived risk was an important rationale for restraint. Fewer participants cited the risk of sexual violence perpetrated by men with schizophrenia as a reason for restraint. Whilst some caregivers referred to specific instances of violence (for example, a relative throwing stones at the neighbours), community leaders and health workers tended to refer to a hypothetical risk. A religious leader and one man with schizophrenia emphasised that aggression by people with mental illness tended to be triggered by the actions of others. The risk of an individual with mental illness damaging property was often cited.

There was considerable pressure on families from the community to act responsibly by restraining their relatives with schizophrenia. One religious leader commented:

*“Of course, it [restraint of people with mental illness] is compulsory. For example, take a dog which is capable of biting. If a person doesn’t tie up a dog, if he accidentally bites people, what would people say? The person will be sued for letting their dog bite someone, now … if a crazy person is not tied up, if he is not held back and if he causes damage on another person, it is another problem, a complaint might come to the people, they might be held accountable for it” *(IV13, Orthodox Christian leader).

On the other hand another religious leader stated that the community would not condone restraint. In the same vein, two caregivers reported that they felt they had no choice but to keep their children restrained, despite the requests of the wider community to unchain them. One explained,

“*My older daughter has leg chains; it’s like the ones they use in the police station. She is very hard to handle; even three to four men can’t handle her. So we chained her up; we gave her the medications by force. She got chained in a shackle with the keys at the back of it for 2 to 3 days. Unless that is done she would break her way out of the walls….Everyone says I should throw the shackles away; they say that there are good children; that they’ve never even seen out. I tell them that I am lost for ways to deal with this and that’s why I’m keeping the shackles. It’s been 2 weeks since she’s left the house*.”

(PRIME FGD05, female caregiver).

#### Punishment

In some cases the threshold for being restrained was reported to be low. Getting into an argument or insulting people would mean people with schizophrenia were assumed to be unwell, and therefore needing to be restrained, although in some cases the ultimate aim of restraint was still to access healthcare. One man with schizophrenia conveyed a stifling need to maintain propriety to avoid being restrained.

“*I don’t get in a fight with people. If I get the chance, I work. If I don’t I just sit. I do not touch people, I also do not touch other people’s property. If I do, they tie me and bring me here [to the health centre]. If I quarrel with people or say bad things to people, they say that I am sick, tie me and bring me here*” (IV06, man with schizophrenia).

In a few cases the rationale for restraint, and physical violence, was more overtly a desire to punish the individual for bad behaviour, for example after having been in a fight with others or damaging property. One male caregiver reported:

“*He set fire to his three suits and his school report card… Now he has got nothing. Then he was tied. After I beat him, he behaved. He doesn’t steal or burn properties*.” (IV03, male caregiver).

Other participants, particularly community leaders, remarked on the need to avoid gratuitous violence or cruelty, and the herbalist suggested this was an outdated approach.

## Discussion

### Summary of findings

The families of people with schizophrenia in rural Ethiopia have a role that is both inherently powerful, yet also paradoxically powerless. The family is powerful in that, unlike in high-income countries, the responsibility for caring for people with mental illness is often entirely in their hands. This study has shown that caregivers often take on the burden of care, and are therefore usually the decision-makers regarding restraining and unrestraining their family members. Restraint was usually described as a pragmatic action, seen as a strategy to manage the illness, based on protecting the person with schizophrenia or the wider community, or was felt to be necessary in order to access treatment. At the same time, families were portrayed largely as being powerless, with extremely limited options and often driven to act out of fear. Lack of accessible and affordable treatment options seemed to underlie much of the caregivers’ narratives. Whilst stigma may not have been discussed overtly, its presence was indicated by the assumption that people with schizophrenia are likely to be violent, and therefore need to be chained.

### Comparison of findings

This study is one of only a small number of reports [15, 19, 40, 41] that focus on restraint of individuals with mental disorders in private community settings in LMIC, rather than wholly or partly on traditional or spiritual healing centres [6, 16, 23] or mental health institutions [18, 20]. With one exception, participants in this study experienced or discussed restraint in the family home. The core drivers of restraint reported by participants accorded with the findings of most previous studies that considered community settings [6, 15, 16, 19, 40, 41]. Conceptualising restraint as a component of care, in settings with few formal mental health services and in particular lack of access to anti-psychotic medication, has been emphasised previously in studies from Ghana [6] and India [40], as well in the context of Ethiopia [22]. The perceived risk of violence posed by people with severe mental illness and a desire to protect the welfare of the individual were familiar themes [6, 15, 16, 19, 41], along with the specific issue of transporting individuals to health facilities [6, 16]. Read et al. have also described the relevance to restraint of emotional and financial burden on caregivers [6]. Similar to other reports the rationale for restraint seemed to be underpinned by stigmatising attitudes towards people with mental illness in some cases [40], in particular the notion of the loss of personhood [6].

There were some notable differences in experiences and drivers of restraint compared to research from other settings. The confinement at home of people with mental illness in India has been described as a ‘zone of social abandonment’ [42], which is “social death, where those who have no place in the social world, yet who are living, are left until they die” [40]. This portrayal is in contrast to our findings from Ethiopia, where people with mental illness appear more likely to be intermittently chained and then unchained as caregivers try to negotiate competing pressures and needs, rather than follow a simple narrative of abandonment. Furthermore, with some exceptions, in this study restraint was not overtly related to “spiritual and moral understandings of the person and society”. For example there was minimal reference to beating out evil spirits or using restraint as a form of punishment [6]. Nor was much weight placed on the need to protect the family from shame by hiding the individual [40] or to prevent substance abuse [6].

### Human rights violations

This study shows that restraint of persons with severe mental illness in Ethiopia results in violation of various human rights enshrined in the CRPD. First, there is a loss of dignity, which is a cornerstone of human rights law (CRPD Article 1), inherent in being physically restrained. There is also clear evidence that the right of all persons to be free from exploitation, violence and abuse (CRPD Article 16) is routinely infringed. Despite not being specifically addressed in the interviews, there are indications that restraint is a discriminatory practice targeted at people with severe mental illness (CRPD Article 5), rather than something carried out on other people who are demonstrating the same behaviours. However, although taking place on a background of stigma, the practice of physical restraint did not seem to be related to lack of awareness about mental illness per se [23].

Article 25 (f) of the CRPD states the importance of ensuring equal access to healthcare for people with disabilities. There are no explicit examples from this data that lack of access to healthcare is a consequence of restraint or that people with mental illness are routinely denied food or water. Conversely, it seems that lack of access to adequate health care leads to the practice of restraint in the community. Restraint is more likely to be aimed at improving access to health services by enabling the safe transportation of people with schizophrenia to the health facility, or by using restraint to ensure they take their medication. What is not clear from the available data is whether people with schizophrenia are typically restrained despite accessing care for their mental illness. It should be borne in mind that in this setting even where mental health care is available, it is often limited to a narrow range of psychotropic medications, with little or no psychosocial support or respite facilities.

Article 12.2 of the CRPD states that “persons with disabilities [should] enjoy legal capacity on an equal basis with others in all aspects of life”. The practice of restraint itself is the most vivid example that people with mental illness are not considered to have legal capacity. In addition there were two instances of using restraint to force the individual to take medication. In a different example, one community leader claimed that people with mental illness would not face the same legal consequences if they committed a crime. There is little data on the violation of other rights such as the respect for family life, education, and employment. However it can be inferred from the sometime long periods that individuals were restrained that individuals may not be able to fully realise those rights.

A fundamental feature of physical restraint is loss of liberty. There are different interpretations of the CRPD Article 14 (1b), which says that persons with disability should not be “deprived of their liberty unlawfully or arbitrarily…and that the existence of a disability shall in no case justify a deprivation of liberty”. There is little controversy about whether arbitrary detention or treatment should be allowed, but rather what constitutes ‘arbitrary’ [43]. Although caregivers had their own ‘rationale’ for restraint, it is important to note that persons with mental illness perceived it as arbitrary. For example one person with schizophrenia reported that he was restrained if he quarrelled with other people.

Recent CRPD committee guidelines state that people with mental disabilities should never be involuntarily detained for treatment, let alone restrained in community settings, even in the circumstances of risk to self or others [44], a view supported by disability and mental health consumer groups [45–47]. The alternative view is that in some circumstances involuntary hospital admission, treatment and even physical restraint may be necessary in psychiatric institutions [48]. A recent systematic review found that physical restraint is included in recommendations for psychiatric emergency care in several LMIC, though with caveats [49].

In an apparent parallel to assessments undertaken by mental health professionals in high-income countries [50], families in rural Ethiopia weigh up the risk to their relative and the community, whilst considering the urgency and need for access to treatment. In the absence of any professional input, family members are left to make this assessment and to effect the action deemed necessary. There is a further parallel in that the general public in high-income countries usually expect that people in need will receive care, and that the public will be protected from harm. This study shows that in rural Ethiopia there is a strong expectation from the community that those responsible for the individual (the family) will take the actions needed to protect the person and the community. Whilst these parallels do not justify the use of restraint, we should note the resemblance to forms of treatment widely accepted in high-income countries, such as involuntary admission and treatment, if played out by different actors.

However, the actions of families in this study appeared to weigh more towards protecting society than individual freedom. Although the potential psychological and physical harm related to restraint was discussed, participants did not frame restraint explicitly as a human rights concern. In the study setting, the priorities and prevailing beliefs focused on the safety and needs of the community or family [6, 22], whilst individuals’ needs were considered chiefly in terms of ensuring they access care irrespective of their wishes [51]. Read et al. have previously noted the contrast between international outrage at the issue of restraint and the mundane way it is sometimes discussed in the communities where it occurs [6]. The fact that all participants, with one exception, acknowledged and were willing to discuss the issue indicates that for the participants of this study restraint is an acceptable, even inevitable, practice. This is arguably consistent with the social and cultural environment in Ethiopia in which patriarchal power relationships exist within families; between community elders and families; and between with medical professionals and families. These power relationships tend to extend to decision-making in relation to care, with the views of the person with mental illness often given less weight than those of health professionals or family members, if they are considered at all.

### Implications for policy and practice

The key implication of this study is that the scale up of accessible and affordable community-based mental healthcare is urgently needed in LMIC, and may go some way to address the issue of restraint [1, 3, 15, 52]. In this study all types of participants cited increasing access to treatment as the most effective way to reduce the incidence of restraint. Mental health care in Ethiopia has historically been centralised in urban settings. The 2012 Ethiopian National Mental Health Strategy signifies a commitment to scaling-up access to mental healthcare [53]. Linked to this, the WHO’s mental health Gap Action Programme, which guides the integration of mental health into primary care, is being piloted and evaluated in several sites across the country [30, 54]. Our study indicates that ensuring that mental health services are locally available in the most remote areas may reduce the reported necessity for restraint to travel to health services. Social health insurance, which is planned for Ethiopia [55], or even free antipsychotic medication, could also have a powerful impact.

The Ethiopian Ministry of Health has stated a commitment to developing and implementing laws to protect the rights of people with mental health problems [53]. Mental health legislation, developed with the involvement of service users [2, 56], is urgently needed in Ethiopia. Although caregivers are the “violators” according to human rights discourse, the lack of agency expressed by this group in our study suggests that legislation should focus on the government’s responsibilities rather than criminalising family members of people with mental illness [6]. Alienating caregivers could result in people with schizophrenia being left without any form of support and vulnerable to vagrancy and premature death [9, 22]. There is also a need for evidence-based guidelines consistent with human rights law for the management of psychiatric emergencies in LMIC, in both inpatient and primary care settings (where most people with mental illness present in this setting [57]) [5, 49].

Physical restraint is a problem unlikely to be addressed adequately by simply providing facility-based mental health services. Even where new models of care are being piloted in Ethiopia the focus is mainly on the provision of psychotropic medication and basic psychoeducation rather than any outreach or rehabilitation work [30]. Instead, a comprehensive package is needed, including support for caregivers, a development approach to encourage economic independence, a concerted effort to encourage uptake of treatment and education to the wider community to reduce stigma [58]. Furthermore family members could be trained to identify triggers for difficult behaviour, as well as techniques to de-escalate violence. As part of the RISE project, CBR is delivered to people with schizophrenia and their caregivers at their home by a specialist CBR worker [31]. The intervention comprises psycho-education, adherence support, rehabilitation (including self-care and social skills), and support accessing existing community organisations. In addition CBR workers conduct community awareness-raising and mobilisation of community support. The effectiveness of CBR on a range of outcomes (including restraint) is being evaluated in the RISE cluster-randomised trial (NCT02160249) [59]. CBR participants and their families receive information about human rights, including guidance on how to avoid physical restraint. CBR workers also discuss how to restrain humanely by, for example, ensuring individuals are in a sheltered place and given sustenance as normal. By including this we are acknowledging that despite the best efforts of all involved, in some circumstances caregivers might feel that restraint is the only option. This element of the intervention highlights some of the challenges in tackling the issue of restraint: arguably by providing this guidance the CBR workers become complicit in the process of restraint, the restraint is normalised and so continues. However, the underlying aim of CBR is to galvanize wider family and community support to enable the caregiver to cope better, to facilitate access to treatment and ultimately to ensure the restraint is not necessary.

There is little empirical evidence on the effectiveness of targeted measures to reduce restraint in community settings. In Indonesia the Bebas Pasung (Free from Restraints) programme involves the provision of community-based mental health services alongside intensive education campaigns [60]. In Somalia the Chain Free Initiative, supported by the WHO, aims to reduce the number of people restrained in hospital and community settings partly through increasing access to mental healthcare [61]. In China, 271 people with mental illness and restrained at home were identified by the Chinese “686 program”. After receiving a package of interventions including “unlocking” by a team of mental health professionals, admission to a psychiatric hospital where required, and follow up by a community mental health team, 92% of patients remained unrestrained after 7 years of follow up [19]. Such an intensive programme is not generalizable to countries such as Ethiopia with highly limited mental health resources and where free healthcare is not available.

### Limitations

This study has some limitations. First, this was a small sample that may not shed light on the full range of experiences of restraint in terms of duration, pattern or reasons. For example, all participants had some access to treatment; it may not be possible to generalise the findings to those who have never accessed care. People experiencing long periods of restraint or confinement are likely to have been inaccessible to the research team. This may explain why the reported pattern of restraint was sometimes focused on short periods of restraining and unrestraining. Second, understanding restraint was not the primary aim of data collection; hence one person with schizophrenia had no personal experience of restraint. Third, there may have been social desirability in all types of participants against discussing overtly abusive practices, neglect or long term restraint. Through involvement in PRIME, some community leaders had been sensitised to the importance of promoting dignity in the care of people with mental illness. This may have skewed the responses of this group and may explain why one community leader denied the practice existed. Caregivers may also have been reluctant to relate restraint to feeling shameful of their family member; practical considerations may have been more acceptable to discuss. Fourth, whilst there was a clear picture of distress experienced by family members involved in restraining, there was little data on how restraint affects how the person affected thinks or feels. Furthermore, little sense emerged of the impact of restraint on the relationship between the person with schizophrenia and their caregiver. Finally, despite an equal split of men and women with schizophrenia in this study, due to the small sample size it was difficult to draw conclusions on the impact of gender on patterns and reasons for restraint. A previous cohort study in this district found an unusually low female to male ratio amongst people identified in the community with severe mental illness [8]. It has been hypothesised that women with psychosis tend to be hidden or confined, therefore not visible to researchers.

## Conclusion

In this study set in rural Ethiopia, most people with schizophrenia and their caregivers had personal experience of the practice of restraint. The main reasons for restraint identified by respondents were to protect the individual or the community, and to access health services. Lack of awareness about mental illness did not emerge as a primary reason for restraint. Restraint entails the violation of several human rights, but the overriding human rights issue is one of lack of access to treatment. The scale up of accessible, affordable and rights based mental health care may go some way to reduce use of restraint in the community. Human rights violations should be monitored in the context of scaling up mental health services to determine whether additional input is required to address the problem.

## Abbreviations

CBR: Community-based rehabilitation
CRPD: Convention on the Rights of Persons with Disabilities
FGD: Focus Groups Discussion
IDI: In-depth interview
LMIC: Low and middle-income countries
MI: Principles Principles for the Protection of Persons with Mental Illness
PRIME: PRogramme for Improving Mental healthcarE
RISE: Rehabilitation Intervention for people with Schizophrenia in Ethiopia
WHO: World Health Organisation
